# Randomized Trial of Finerenone on Urinary Albumin-to-Creatinine Ratio in Type 2 Diabetes Mellitus and CKD

**DOI:** 10.1016/j.ekir.2026.106546

**Published:** 2026-04-16

**Authors:** Atsushi Tanaka, Takumi Imai, Muthiah Vaduganathan, Yosuke Okada, Satomi Sonoda, Keiichi Torimoto, Satoru Suwa, Hiroki Teragawa, Motoaki Miyazono, Makoto Fukuda, Keisuke Yonezu, Naohiko Takahashi, Yuichi Yoshida, Kenichi Tanaka, Michio Shimabukuro, Yuki Hotta, Masao Moroi, Hiroki Niikura, Keisuke Kida, Kenichi Yokota, Daiju Fukuda, Kengo Tanabe, Yu Horiuchi, Shigeru Toyoda, Isao Taguchi, Hisako Yoshida, Toru Miyoshi, Masaomi Nangaku, Hirotaka Shibata, Koichi Node, FIVE-STAR Investigators

**Affiliations:** 1 Department of Cardiovascular Medicine, Saga University, Saga, Japan; 2 Clinical Research Division, Organization for Clinical Medicine Promotion, Tokyo, Japan; 3 Clinical and Translational Research Center, Kobe University Hospital, Kobe, Japan; 4 Division of Cardiovascular Medicine, Brigham and Women's Hospital, Harvard Medical School, Boston, Massachusetts, USA; 5 Clinical Research Center, Hospital of the University of Occupational and Environmental Health, Japan, Kitakyushu, Japan; 6 First Department of Internal Medicine, University of Occupational and Environmental Health, Japan, Kitakyushu, Japan; 7 Department of Cardiology, Juntendo University Shizuoka Hospital, Izunokuni, Japan; 8 Department of Cardiovascular Medicine, JR Hiroshima Hospital, Hiroshima, Japan; 9 Department of Nephrology, Saga University, Saga, Japan; 10 Department of Cardiology and Clinical Examination, Faculty of Medicine, Oita University, Yufu, Japan; 11 Department of Endocrinology, Metabolism, Rheumatology and Nephrology, Faculty of Medicine, Oita University, Yufu, Japan; 12 Wakamatsu Hospital of the University of Occupational and Environmental Health, Japan, Kitakyushu, Japan; 13 Department of Diabetes, Endocrinology, and Metabolism, Fukushima Medical University School of Medicine, Fukushima, Japan; 14 Division of Cardiovascular Medicine, Toho University Ohashi Medical Center, Tokyo, Japan; 15 Department of Pharmacology, St. Marianna University School of Medicine, Kawasaki, Japan; 16 Division of Metabolism and Endocrinology, Department of Internal Medicine, St. Marianna University School of Medicine, Kawasaki, Japan; 17 Department of Cardiovascular Medicine, Osaka Metropolitan University Graduate School of Medicine, Osaka, Japan; 18 Division of Cardiology, Mitsui Memorial Hospital, Tokyo, Japan; 19 Department of Cardiovascular Medicine, Dokkyo Medical University, Mibu, Japan; 20 Department of Cardiology, Dokkyo Medical University Saitama Medical Center, Koshigaya, Japan; 21 Department of Medical Statistics, Osaka Metropolitan University Graduate School of Medicine, Osaka, Japan; 22 Department of Cardiovascular Medicine, Okayama University Graduate School of Medicine, Dentistry and Pharmaceutical Sciences, Okayama, Japan; 23 Division of Nephrology and Endocrinology, The University of Tokyo Graduate School of Medicine, Tokyo, Japan

**Keywords:** chronic kidney disease, combination therapy, finerenone, glucagon-like peptide-1 receptor agonist, sodium-glucose cotransporter 2 inhibitor, urinary albumin excretion

## Introduction

Finerenone improved kidney and cardiovascular outcomes in patients with type 2 diabetes (T2D) and chronic kidney disease (CKD) when coadministered with conventional renin-angiotensin system inhibitors (RASi).^1^ A mediation analysis using pooled data from placebo-controlled kidney and cardiovascular outcomes trials of finerenone in 12,512 patients with T2D and CKD revealed that early reduction in urinary albumin excretion largely explains finerenone’s therapeutic benefits in kidney outcomes.^2^

Finerenone, together with RASi, sodium-glucose cotransporter 2 inhibitors (SGLT2i), and glucagon-like peptide-1 receptor agonists (GLP-1RA), are now considered “pillars of care” for delaying CKD progression in patients with T2D and CKD.^3^ However, clinical data on the efficacy and safety of finerenone by combination medication status remain limited. In this secondary analysis of the FIVE-STAR trial,^4^ we aimed to assess the effects of finerenone on urinary albumin excretion according to background therapy (RASi, SGLT2i, and GLP-1RA) status in Japanese patients with T2D and CKD.

Detailed methods are described in the Supplementary Methods section.

## Results

### Patient Characteristics

Among the participants included in the full-analysis dataset of the FIVE-STAR study (*N* = 101),^4^ the present analysis included 97 patients with available urinary albumin-to-creatinine ratio (UACR) data (Supplementary Figure S1). Baseline characteristics were generally balanced between the arms (Supplementary Table S1). The median estimated glomerular filtration rate (eGFR) was 56.2 ml/min per 1.73 m^2^, and median UACR was 193.8 mg/g. The proportion of patients using the medications of interest at baseline was 79.4%, 62.9%, and 30.9% for RASi, SGLT2i, and GLP-1RA, respectively. Overall, 35.1% of the patients were on no therapy or single-drug regimens, 49.5% were on 2 therapies, and 15.5% were on all 3 therapies. Within this subanalysis, among the 92 patients receiving treatment at week 24, 80 patients (87.0%; 39 [83.0%] on finerenone; 41 [91.1%] on placebo) were on the target dose (20 mg) of the study drugs at week 24 (Supplementary Table S2). No patients experienced new initiation of the medications of interest or intensification of treatment regimens during the follow-up period (Supplementary Table S3).

### UACR Change by Background Medication Status

**Figure 1:**
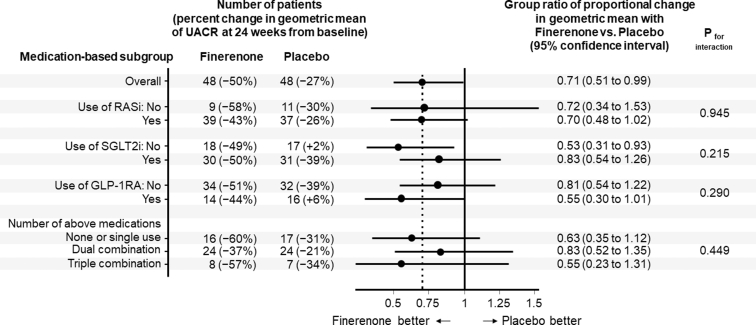
UACR response by background medication status. Of the 97 participants with UACR follow-up data, 96 (48 in each treatment group) had week 24 data. The remaining participant, for whom only week 12 data were available, was also included in the mixed-effects analysis. The *P*-value for interaction was obtained from the treatment × subgroup interaction term at week 24. GLP-1RA, glucagon-like peptide-1 receptor antagonist; RASi, renin-angiotensin system inhibitor; SGLT2i, sodium-glucose co-transporter 2 inhibitor; UACR, urinary albumin-to-creatinine ratio.

As reported previously,^4^ finerenone therapy over 24 weeks caused a 29% greater reduction in UACR levels than that found for placebo in the overall study cohort. The impact of finerenone on urinary albumin was consistent when coadministered with each of the 3 background therapies analyzed and when administered in participants already on dual or triple combination regimens (Figure 1).

### Changes in eGFR, Serum Potassium, and Blood Pressures

**Figure 2:**
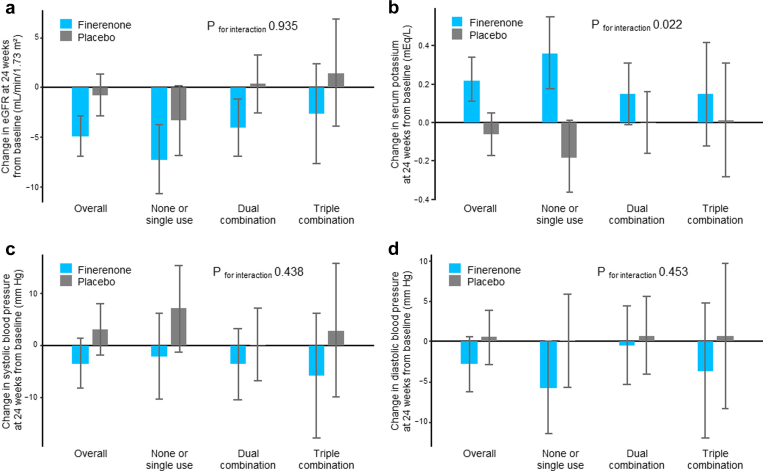
Changes in eGFR (a), serum potassium (b), and blood pressures (c for systolic and d for diastolic) by background medication status (none or single use, dual combination, and triple combination). The *P*-value for interaction was obtained from the treatment × subgroup interaction term at week 24. eGFR, estimated glomerular filtration rate.

The background medication status did not affect the changes in the eGFR and blood pressures (Figure 2a, c, and d, and Supplementary Table S4). Conversely, finerenone therapy caused a greater increase in serum potassium compared with placebo in the ‘none or single use’ subgroup than that in the other medication subgroups (Figure 2b). Furthermore, the treatment effect on changes in the serum potassium levels over 24 weeks differed among background medication status; and further between SGLT2i users and nonusers (Supplementary Table S4).

## Discussion

This secondary analysis of the FIVE-STAR study demonstrated consistent reductions in UACR, an integrative marker of cardio-kidney health, with finerenone across a broad range of background therapies of interest among Japanese patients with T2D and CKD.

The therapeutic landscape of T2D and CKD has dramatically evolved recently owing to the identified potential of SGLT2i, GLP-1RA, and finerenone, promoting their use as potential “pillars of care” and as combination regimens in patients with T2D and CKD.^3^ Cross-trial analyses have suggested that combining SGLT2i, GLP-1RA, and finerenone could delay CKD progression and improve cardiovascular- and kidney event-free survivals in patients with T2D and elevated albuminuria.^5^

Notably, the participants in the FIVE-STAR study were better treated with disease-modifying therapies (62.9% taking SGLT2i, 30.9% taking GLP-1 RA) at baseline than those in the FIDELITY study (6.7% taking SGLT2i, 7.2% taking GLP-1RA).^1^ This may partially explain the substantial decrease (27%) in UACR even in the placebo group in this study cohort. Furthermore, the proportion of background RASi use (79%) for patients in the FIVE-STAR cohort was lower than that in the trials; however, the results are comparable to those of a recent real-world database study in Japan.^6^ Thus, a key strength of the present analysis includes its possible reflection of the real-world clinical setting at least in Japan.

The present findings of consistent UACR reduction across background therapeutic regimens are partially similar to those of previous randomized evidence from the CONFIDENCE trial which directly assessed the effects or combined finerenone and SGLT2i against either medication alone on the UACR trajectory in patients with T2D and CKD.^7^ Our findings are also comparable to the more recent FIDELITY *post hoc* analysis assessing the effects of finerenone when taken with concomitant SGLT2i and GLP-1RA.^8^ These similarities emphasize the need to establish better approaches for implementing combinations of the 4-pillar medications to achieve optimal therapeutic effects against T2D and CKD.^3^

This study showed that eGFR and blood pressures changes in response to finerenone were consistent by background medication status. The effects of finerenone on increasing the serum potassium levels appeared to be attenuated among those treated with combination therapies, particularly in those with concomitant SGLT2i. This may partially explain the numerically lower hyperkalemia in the combination therapy group (finerenone plus SGLT2i) than that in the finerenone alone group observed in the CONFIDENCE and FIDELITY trials.^7^^,^^8^ In a more recent secondary analysis of the CONFIDENCE trial,^9^ changes in serum potassium levels were statistically similar between finerenone plus empagliflozin and finerenone alone groups. This is inconsistent with our findings; the underlying reasons for this discrepancy are unclear. A key difference between the CONFIDENCE trial and this study involves the method of initiating the combination therapy of finerenone and SGLT2i. Our findings suggest that the chronic administration of background SGLT2i may have attenuated the increase in serum potassium levels caused by add-on finerenone therapy. However, the effects of SGLT2i on serum potassium levels and their mechanisms remain unelucidated. Hence, further experimental and clinical validations are required to develop and investigate hypotheses regarding these effects.

This study had some limitations. First, the FIVE-STAR study included a relatively small sample size and was not primarily designed for the subgroup analyses conducted in this study. Data obtained from analyses with small sample sizes across different therapy groups and with limited statistical power should be interpreted cautiously. Additionally, all analyses were performed at a 2-sided significance level of 0.05 without adjustment for multiplicity, potentially increasing the probability of chance findings. Second, we did not investigate detailed information on the background medications, such as individual doses, usage duration before study enrollment, and changes and adherence during follow-up. Additionally, the participants in each group were not stratified according to those factors. Third, the follow-up period was short and was not sufficient to evaluate the long-term effects of finerenone on renal function with different baseline therapy. Analysis of the long-term eGFR slope is required to elucidate the effects on longitudinal CKD progression. Finally, the study included only Japanese participants, limiting the generalizability of the findings.

In conclusion, this study showed that the impact of finerenone on urinary albumin levels was consistent regardless of the background concomitant medications, including RASi, SGLT2i, and GLP-1RA, in Japanese patients with T2D and CKD.

## Disclosure

AT received honoraria from Boehringer Ingelheim Japan, Mochida, Otsuka, Novo Nordisk, and Amgen and research funding from Bristol Myers Squibb. TI has received lecture fees from Eizai Pharmaceutical and has served as a statistician in the Organization for Clinical Medicine Promotion. MV has received research grant support, served on advisory boards, and has had speaker engagements with Alnylam Pharmaceuticals, American Regent, Amgen, AstraZeneca, Bayer AG, Baxter Healthcare, BMS, Boehringer Ingelheim, Chiesi, Cytokinetics, Esperion, Fresenius Medical Care, Idorsia Pharmaceuticals, Lexicon Pharmaceuticals, Merck, Milestone Pharmaceuticals, Novartis, Novo Nordisk, Pharmacosmos, Recordati, Relypsa, Roche Diagnostics, Sanofi, and Tricog Health, and has participated in clinical trial committees for studies sponsored by Amgen, AstraZeneca, Bayer AG, Boehringer Ingelheim, Galmed, Impulse Dynamics, Novartis, Occlutech, and Pharmacosmos. SSuwa received honoraria from Abbott Medical Japan and Daiichi Sankyo. HT received honoraria from Abbott Medical Japan, Bayer, Boehringer Ingelheim, Daiichi Sankyo, Kowa, Medtronic, and Nihon Medi-Physics. MMiyazono received honoraria from Chugai Pharmaceutical, Astellas, Kyowa Kirin, Mitsubishi Tanabe, Mochida Pharmaceutical, AstraZeneca, Bayer Yakuhin, Torii Pharmaceutical, Fuso Pharmaceutical Industries, Daiichi Sankyo Company, Limited, Teijin Pharma, Eli Lilly Japan K.K., Nippon Boehringer Ingelheim Co., Ltd., Kissei Pharmaceutical, and Kowa Company, Ltd., research funding from Chugai Pharmaceutical and Kyowa Kirin, and scholarships from Kyowa Kirin, Torii Pharmaceutical, Fuso Pharmaceutical Industries, and Kissei Pharmaceutical. NT received honoraria from Daiichi Sankyo, Novartis Pharma, Bristol Myers Squibb, Pfizer, Otsuka Pharmaceutical, Medtronic, Toa Eiyo, and Nippon Boehringer-Ingelheim. MS received honoraria from Bayer Yakuhin, Ltd. MMoroi received honoraria from Sanofi, Amicus Therapeutics, Takeda, Otsuka, Kowa, Mitsubishi Tanabe Pharma, Daiichi Sankyo, AstraZeneca, Nihon Medi-physics, and Bayer and research funding from Taisho Pharmaceutical. KK received honoraria from AstraZeneca K.K., Ono Pharmaceutical Co., Ltd., Nippon Boehringer Ingelheim Co., Ltd., Bayer Yakuhin, Ltd., Otsuka Pharmaceutical Co., Ltd., and Novartis Pharmaceuticals Co., Ltd. DF received research funding from AMGEN Inc. and BIOTRONIK Japan, Inc. and honoraria from Novartis Pharma K.K., Otsuka Pharmaceutical Co., Ltd., and Kowa Company, Ltd. KTanabe received honoraria from Kowa, Boehringer-Ingelheim, Daiichi Sankyo, Toa Eiyo, Eli Lilly, Novartis, Novo Nordisk, Bayer, Pfizer, AstraZeneca, Ono pharma, Otsuka, and Takeda. YHoriuchi received honoraria from Bayer Pharmaceuticals and research funding from Asahi Intecc. HY received outsourcing fees from the organization for Clinical Medicine Promotion. TM received honoraria from Kowa, Novartis Japan, and AstraZeneca and research funding from Kowa. MN received honoraria and advisory fees from Boehringer Ingelheim, Kyowa Kirin, Astellas, Astra Zeneca, GSK, Daiichi Sankyo, Tanabe-Mitsubishi, Chugai, Torii, JT, and Novo Nordisk and research funding from Kyowa-Kirin, Daiichi Sankyo, Astellas, Ono, Tanabe-Mitsubishi, JT, Chugai, Bayer, Torii, and Takeda. HS received honoraria from Bayer and Daiichi Sankyo, Japan. KN has received honoraria from MSD, AstraZeneca, Novartis, Novo Nordisk, Bayer, Kowa, Mochida, Otsuka, Daiichi Sankyo, Mitsubishi Tanabe, Eli Lilly Japan, and Boehringer Ingelheim Japan, research grant from Fujiyakuhin, Astellas, Novartis, Bayer, and Mochida, and scholarship from Abbott Medical, Daiichi Sankyo, Mitsubishi Tanabe Pharma, Teijin Pharma, and Boehringer Ingelheim Japan. All other authors declare no competing interests.

## Supplementary Material

Supplementary File (PDF) **Supplementary Methods**. **Supplementary Results**. **Figure S1.** CONSORT diagram. **Table S1.** Background patient characteristics. **Table S2.** Doses of study drugs in on-treatment patients at week 24 by baseline medication status. **Table S3.** Change in frequency of concomitant medications of interest at week 24. Table S4. Changes in eGFR, serum potassium, and blood pressures by background medication status.
