# Supplementary material for: Randomized Trial of Finerenone on Urinary Albumin-to-Creatinine Ratio in Type 2 Diabetes Mellitus and CKD
Source: Kidney Int Rep. 2026 Apr 16;11(7):106546. doi: 10.1016/j.ekir.2026.106546 (PMC13195771; doi:10.1016/j.ekir.2026.106546)
Supplement: Supplementary File (PDF) — Supplementary Methods. Supplementary Results. Figure S1. CONSORT diagram. Table S1. Background patient characteristics. Table S2. Doses of study drugs in on-treatment patients at week 24 by baseline medication status. Table S3. Change in frequency of concomitant medications of interest at week 24. Table S4. Changes in eGFR, serum potassium, and blood pressures by background medication status. [file mmc1.pdf]

Supplementary Material

Randomized Trial of Finerenone on UACR in T2 and CKD

Atsushi Tanaka[1], Takumi Imai[2,3], Muthiah Vaduganathan[4], Yosuke Okada[5], Satomi Sonoda[6], Keiichi Torimoto[6], Satoru Suwa[7], Hiroki Teragawa[8], Motoaki Miyazono[9], Makoto Fukuda[9], Keisuke Yonezu[10], Naohiko Takahashi[10], Yuichi Yoshida[11], Kenichi Tanaka[12], Michio Shimabukuro[13], Yuki Hotta[13], Masao Moroi[14], Hiroki Niikura[14], Keisuke Kida[15], Kenichi Yokota[16], Daiju Fukuda[17], Kengo Tanabe[18], Yu Horiuchi[18], Shigeru Toyoda[19], Isao Taguchi[20], Hisako Yoshida[21], Toru Miyoshi[22], Masaomi Nangaku[23], Hirotaka Shibata[11], Koichi Node[1], on behalf of the FIVE-STAR Investigators.

[1]Department of Cardiovascular Medicine, Saga University, Saga, Japan; [2]Clinical Research Division, Organization for Clinical Medicine Promotion, Tokyo, Japan; [3]Clinical and Translational Research Center, Kobe University Hospital, Kobe, Japan; [4]Division of Cardiovascular Medicine, Brigham and Women's Hospital, Harvard Medical School, Boston, MA, USA; [5]Clinical Research Center, Hospital of the University of Occupational and Environmental Health, Japan, Kitakyushu, Japan; [6]First Department of Internal Medicine, University of Occupational and Environmental Health, Japan, Kitakyushu, Japan; [7]Department of Cardiology, Juntendo University Shizuoka Hospital,

Izunokuni, Japan; [8]Department of Cardiovascular Medicine, JR Hiroshima Hospital, Hiroshima, Japan; [9]Department of Nephrology, Saga University, Saga, Japan; [10]Department of Cardiology and Clinical Examination, Faculty of Medicine, Oita University, Yufu, Japan; [11]Department of Endocrinology, Metabolism, Rheumatology and Nephrology, Faculty of Medicine, Oita University, Yufu, Japan; [12]Wakamatsu Hospital of the University of Occupational and Environmental Health, Japan, Kitakyushu, Japan; [13]Department of Diabetes, Endocrinology, and Metabolism, Fukushima Medical University School of Medicine, Fukushima, Japan; [14]Division of Cardiovascular Medicine, Toho University Ohashi Medical Center, Tokyo, Japan; [15]Department of Pharmacology, St. Marianna University School of Medicine, Kawasaki, Japan; [16]Division of Metabolism and Endocrinology, Department of Internal Medicine, St. Marianna University School of Medicine, Kawasaki, Japan; [17]Department of Cardiovascular Medicine, Osaka Metropolitan University Graduate School of Medicine, Osaka, Japan; [18]Division of Cardiology, Mitsui Memorial Hospital, Tokyo, Japan; [19]Department of Cardiovascular Medicine, Dokkyo Medical University, Mibu, Japan; [20]Department of Cardiology, Dokkyo Medical University Saitama Medical Center, Koshigaya, Japan; [21]Department of Medical Statistics, Osaka Metropolitan University Graduate School of Medicine, Osaka, Japan; [22]Department of Cardiovascular

Medicine, Okayama University Graduate School of Medicine, Dentistry and Pharmaceutical Sciences, Okayama, Japan; [23]Division of Nephrology and Endocrinology, The University of Tokyo Graduate School of Medicine, Tokyo, Japan

Correspondence: Atsushi Tanaka, Department of Cardiovascular Medicine, Saga University, 5-1-1 Nabeshima, Saga, 849-8501, Japan. E-mail: tanakaa2@cc.saga-u.ac.jp

Supplementary Methods

Study Design and Procedures

This study was a post-hoc secondary analysis of an investigator-initiated, multicenter, prospective, placebo-controlled, double-blind, randomized clinical trial (FIVE-STAR) conducted in Japan to investigate the effects of 24-week finerenone therapy on cardiovascular- and kidney-related biomarkers in patients with type 2 diabetes (T2D) and chronic kidney disease (CKD) (ClinicalTrials.gov NCT05887817 and jRCTs021230011). The study protocol was approved centrally by the Certified Review Board of Fukushima Medical University (no. F2023001), and the study adhered to the principles of the Declaration of Helsinki and the Clinical Trial Act in Japan. Written informed consent was obtained from all participants. Detailed rationale and original design of the study have been previously published.[4,S1]

After informed consent was obtained, the participants were randomly and equally allocated to receive once-daily oral finerenone or a matching placebo via a web-based dynamic allocation system. Randomization was conducted through a minimization method balancing for age (<70 or ≥70 years), sex, estimated glomerular filtration rate (eGFR; <45 or ≥45 mL/min/1.73 m^2), and sodium-glucose cotransporter 2 inhibitor (SGLT2i) use at the time of consent. The study drug (finerenone) and placebo tablets

supplied by Bayer were identical in appearance to maintain blinding. Participants with a baseline eGFR <60 mL/min/1.73 m^2 started with 10 mg of the study drug, and an up-titration to 20 mg was encouraged after 4 weeks. Participants with a baseline eGFR ≥60 mL/min/1.73 m^2 received initial and maintenance doses of 20 mg. Down-titration and discontinuation of the study drug were allowed according to the patient's medical condition, such as serum potassium and eGFR levels, at the discretion of the local investigator. After the study drug was initiated, patients were required, in principle, not to start new SGLT2i or change the dosage of concomitant medications during the individual interventional period. Study follow-up visits were set at weeks 4, 12, and 24 after the initiation of the study drugs. All patients and trial personnel were blinded to the treatment arms. An electronic data capture system (eClinical Base, Translational Research Center for Medical Innovation, Kobe, Japan) was used for data collection and management, and the retrieved data were monitored independently.

Study Population and Sample Size

The trial included adult participants diagnosed with T2D and CKD, meeting both of the following criteria: an eGFR ≥25 to <90 mL/min/1.73 m^2 and elevated urinary albumin-to-creatinine ratio (UACR, ≥30 to <3500 mg/g.Cr). Participants were required to have

had no changes in T2D and CKD medications within 4 weeks before the consent. Key exclusion criteria were uncontrolled T2D status, serum potassium ≥4.9 mEq/L, symptomatic heart failure with reduced left ventricular ejection fraction, or a recent history of cardiovascular and renal events within 8 weeks before the consent. Participants were recruited at 13 sites in Japan between September 2023 and February 2024.

The trial was primarily designed to evaluate the effect of finerenone relative to that of placebo on vascular stiffness, as assessed by the cardio-ankle vascular index (CAVI) using the VaSera device (Fukuda Denshi, Co., Ltd., Tokyo, Japan). A total of 100 participants (50 patients per study arm) was minimally set to detect the meaningful difference in the CAVI change over 24 weeks between treatment groups at a 5% significance level (two-sided) with 80% power, accounting for a 10% drop-out rate.[S1] The primary results have been published elsewhere.[4]

Among the full analysis set population of the trial (N = 101),[4] 97 participants (49 for the finerenone group and 48 for the placebo group), whose UACR follow-up data were available at baseline and follow-up, were included in the present analysis (**Supplementary Figure S1**).

Outcome Measures

The primary endpoint of the present study was the change in UACR over 24 weeks according to the background status of the medications of interest; renin-angiotensin system inhibitors (RASi; angiotensin-converting enzyme inhibitors, angiotensin receptor blockers, or angiotensin receptor-neprilysin inhibitor), SGLT2i, and glucagon-like peptide-1 receptor agonists (GLP-1RA). Changes in eGFR, serum potassium, and blood pressures over 24 weeks were also assessed as secondary endpoints.

Statistical Analyses

The baseline demographic and clinical characteristics are summarized as medians (interquartile ranges) or counts with percentages. Subgroup analyses were performed for all outcomes according to the (1) use of RASi (no/yes), (2) use of SGLT2i (no/yes), (3) use of GLP-1RA (no/yes), and (4) the number of these concomitant agents used (none or single use, dual combination, or triple combination). For each subgroup factor, a separate mixed-effects model for repeated measures with a compound symmetry covariance structure was fitted. In each model, the fixed effects included treatment group, time (categorical), the subgroup variable of interest, and the two- and three-way interactions between treatment, time and subgroup. The baseline value of the

respective outcome variable was included as a covariate. The between-group differences at 24 weeks (the primary study visit) were estimated based on least-squares means. The P value for interaction was obtained from the treatment × subgroup interaction term at week 24. For UACR, analyses were performed on log-transformed values, and results were presented using geometric means. All statistical analyses were exploratory, and P values were interpreted in a descriptive manner. A two-sided significance level of 0.05 was used without adjustment for multiplicity. All analyses were performed using R (version 4.4.2; R Foundation for Statistical Computing, Vienna, Austria).

Supplementary Results

Supplementary Figure S1. CONSORT diagram

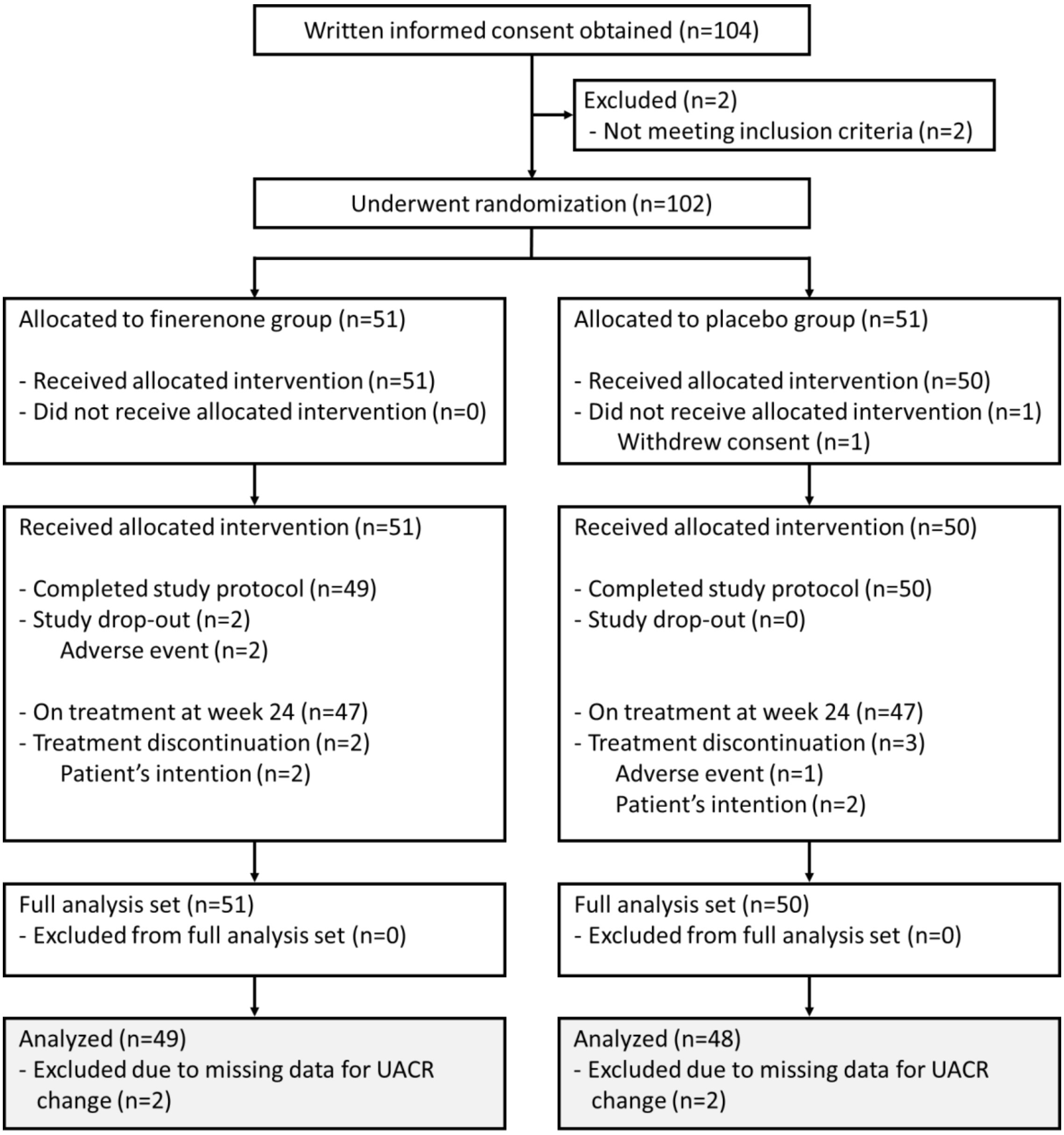

This diagram was modified from the published paper of the primary result of the FIVE-STAR study.[4]

UACR, urinary albumin-to-creatinine ratio

Supplementary Table S1. Background patient characteristics

Variable	Total (n = 97)	Finerenone (n = 49)	Placebo (n = 48)	Standardised mean difference between treatment groups
Age,* years	73 (63, 79)	73 (64, 80)	73 (63, 77)	0.082
Male sex	66 (68.0)	33 (67.3)	33 (68.8)	0.030
Body mass index, kg/m^2	24.4 (22.3, 27.9)	25.0 (21.9, 27.2)	24.4 (22.7, 27.9)	0.169
Systolic blood pressure, mm Hg	129 (120, 143)	128 (122, 144)	129 (119, 142)	0.137
Diastolic blood pressure, mm Hg	74 (65, 83)	74 (64, 82)	74 (67, 84)	0.052
History of hypertension	89 (91.8)	46 (93.9)	43 (89.6)	0.156
History of heart failure	25 (25.8)	13 (26.5)	12 (25.0)	0.035
Duration of diabetes,* years	14.0 (10.0, 23.3)	14.6 (10.1, 24.3)	11.7 (8.1, 22.5)	0.202
HbA1c,* %	6.8 (6.5, 7.4)	7.0 (6.5, 7.4)	6.7 (6.3, 7.3)	0.146
Diabetic nephropathy (etiology of CKD)	80 (82.5)	42 (85.7)	38 (79.2)	0.173
Serum potassium,* mEq/L	4.3 (4.0, 4.6)	4.3 (4.0, 4.5)	4.3 (4.2, 4.6)	0.104
eGFR,* mL/min/1.73 m^2	56.2 (45.2, 66.0)	56.1 (46.4, 64.6)	57.3 (44.2, 66.8)	<0.001
Distribution				
≥60 mL/min/1.73 m^2	42 (43.3)	20 (40.8)	22 (45.8)	0.101

45 to <60 mL/min/1.73 m^2	31 (32.0)	18 (36.7)	13 (27.1)	0.208
30 to <45 mL/min/1.73 m^2	17 (17.5)	8 (16.3)	9 (18.8)	0.064
<30 mL/min/1.73 m^2	7 (7.2)	3 (6.1)	4 (8.3)	0.085
UACR,* mg/g.Cr	193.8 (68.0, 500.6)	221.8 (68.0, 501.0)	190.9 (71.3, 434.1)	0.040
Distribution				
30 to <300 mg/g.Cr	63 (64.9)	31 (63.3)	32 (66.7)	0.071
≥300 mg/g.Cr	34 (35.1)	18 (36.7)	16 (33.3)	0.071
KDIGO risk category distribution				
Moderate	29 (29.9)	13 (26.5)	16 (33.3)	0.149
High	32 (33.0)	18 (36.7)	14 (29.2)	0.162
Very high	36 (37.1)	18 (36.7)	18 (37.5)	0.016
Baseline medications				
Renin-angiotensin system inhibitors	77 (79.4)	40 (81.6)	37 (77.1)	0.113
ACEI	6 (6.2)	4 (8.2)	2 (4.2)	0.167
ARB	57 (58.8)	29 (59.2)	28 (58.3)	0.017
ARNI	15 (15.5)	7 (14.3)	8 (16.7)	0.066
Insulin	14 (14.4)	7 (14.3)	7 (14.6)	0.008
Metformin	39 (40.2)	22 (44.9)	17 (35.4)	0.194
DPP-4 inhibitor	48 (49.5)	26 (53.1)	22 (45.8)	0.145

SGLT2i	61 (62.9)	30 (61.2)	31 (64.6)	0.070
GLP-1RA	30 (30.9)	14 (28.6)	16 (33.3)	0.103
Number of medications†				
None or single use	34 (35.1)	17 (34.7)	17 (35.4)	0.015
Dual combination	48 (49.5)	24 (49.0)	24 (50.0)	0.020
Triple combination	15 (15.5)	8 (16.3)	7 (14.6)	0.048

Values are expressed as median (interquartile range) or n (%).

* At randomization

† RASi, SGLT2i, and GLP-1RA

ACEI, angiotensin converting enzyme inhibitor; ARB, angiotensin receptor blocker; ARNI, angiotensin receptor-neprilysin inhibitor; CKD, chronic kidney disease; DPP-4, dipeptidyl peptidase-4; eGFR, estimated glomerular filtration rate; GLP-1RA, glucagon-like peptide-1 receptor antagonist; KDIGO, Kidney Disease: Improving Global Outcomes; SGLT2i, sodium-glucose cotransporter 2 inhibitor; UACR, urinary albumin-to-creatinine ratio

Supplementary Table S2. Doses of study drugs in on-treatment patients at week 24 by baseline medication status

	Overall		None or single use		Dual combination		Triple combination	
	**Finerenone (*n* = 47)**	**Placebo (*n* = 45)**	**Finerenone (*n* = 16)**	**Placebo (*n* = 16)**	**Finerenone (*n* = 23)**	**Placebo (*n* = 23)**	**Finerenone (*n* = 8)**	**Placebo (*n* = 6)**
At baseline								
10 mg	26 (55.3)	25 (55.6)	7 (43.8)	8 (50.0)	14 (60.9)	13 (56.5)	5 (62.5)	4 (66.7)
20 mg	21 (44.7)	20 (44.4)	9 (56.3)	8 (50.0)	9 (39.1)	10 (43.5)	3 (37.5)	2 (33.3)
At week 24								
10 mg	8 (17.0)	4 (8.9)	2 (12.5)	2 (12.5)	4 (17.4)	1 (4.3)	2 (25.0)	1 (16.7)
20 mg	39 (83.0)	41 (91.1)	14 (87.5)	14 (87.5)	19 (82.6)	22 (95.7)	6 (75.0)	5 (83.3)

Values are expressed as n (%).

Supplementary Table S3. Change in frequency of concomitant mediations of interest at week 24

Pillar Medication	At baseline			At week 24		
	Total (*n* = 97)	Finerenone (*n* = 49)	Placebo (*n* = 48)	Total (*n* = 97)	Finerenone (*n* = 49)	Placebo (*n* = 48)
RASi (ACEI, ARB, or ARNI)	77 (79.4)	40 (81.6)	37 (77.1)	76 (78.4)	39 (79.6)	37 (77.1)
SGLT2i	61 (62.9)	30 (61.2)	31 (64.6)	60 (61.9)	29 (59.2)	31 (64.6)
GLP-1RA	30 (30.9)	14 (28.6)	16 (33.3)	28 (28.9)	14 (28.6)	14 (29.2)
Number of medications*						
None or single use	34 (35.1)	17 (34.7)	17 (35.4)	34 (35.1)	17 (34.7)	17 (35.4)
Dual combination	48 (49.5)	24 (49.0)	24 (50.0)	51 (52.6)	26 (53.1)	25 (52.1)
Triple combination	15 (15.5)	8 (16.3)	7 (14.6)	12 (12.4)	6 (12.2)	6 (12.5)

Values are expressed as n (%).

* RASi, SGLT2i, and GLP-1RA

ACEI, angiotensin converting enzyme inhibitor; ARB, angiotensin receptor blocker; ARNI, angiotensin receptor-neprilysin inhibitor; GLP-1RA, glucagon-like peptide-1 receptor antagonist; RASi, renin-angiotensin system inhibitor; SGLT2i, sodium-glucose cotransporter 2 inhibitor

Supplementary Table S4. Changes in eGFR, serum potassium, and blood pressures by background medication status

Variables/background medication status/time point	Finerenone	Placebo	Inter-group Difference	Inter-group P-value	*P*-value for subgroup interaction*
eGFR, mL/min/1.73 m^2					
Overall	(N = 49)	(N = 48)			
Baseline	58.0 (53.1 to 63.0)	57.0 (52.0 to 62.0)			
Week 24	53.2 (48.2 to 58.1)	56.2 (51.2 to 61.2)			
Δ	−4.88 (−6.92 to −2.85)	−0.78 (−2.84 to 1.28)	−3.99 (−6.81 to −1.18)	0.006	
Use of RASi, No	(N = 9)	(N = 11)			
Baseline	63.6 (52.0 to 75.2)	59.9 (49.4 to 70.4)			
Week 24	57.8 (46.2 to 69.4)	56.7 (46.2 to 67.2)			
Δ	−5.79 (−10.55 to −1.02)	−3.19 (−7.50 to 1.12)	−2.24 (−8.51 to 4.03)	0.482	0.518
Use of RASi, Yes	(N = 40)	(N = 37)			
Baseline	56.8 (51.3 to 62.3)	56.1 (50.4 to 61.8)			
Week 24	52.1 (46.6 to 57.6)	56.1 (50.3 to 61.8)			
Δ	−4.68 (−6.94 to −2.42)	−0.06 (−2.42 to 2.29)	−4.55 (−7.73 to −1.37)	0.005	
Use of SGLT2i, No	(N = 19)	(N = 17)			
Baseline	58.2 (50.1 to 66.2)	58.8 (50.3 to 67.3)			0.531
Week 24	52.0 (44.0 to 60.1)	55.3 (46.9 to 63.8)			

Δ	−6.12 (−9.35 to −2.90)	−3.44 (−6.84 to −0.03)	−2.75 (−7.32 to 1.83)	0.238	
Use of SGLT2i, Yes	(N = 30)	(N = 31)			
Baseline	58.0 (51.6 to 64.4)	56.0 (49.7 to 62.3)			
Week 24	53.9 (47.5 to 60.3)	56.7 (50.4 to 63.0)			
Δ	−4.10 (−6.66 to −1.53)	0.67 (−1.85 to 3.20)	−4.58 (−8.09 to −1.07)	0.011	
Use of GLP-1RA, No	(N = 35)	(N = 32)			
Baseline	58.1 (52.2 to 64.0)	56.1 (49.9 to 62.3)			
Week 24	52.5 (46.6 to 58.4)	55.5 (49.4 to 61.7)			
Δ	−5.60 (−8.03 to −3.17)	−0.55 (−3.09 to 1.99)	−4.85 (−8.27 to −1.43)	0.006	0.349
Use of GLP-1RA, Yes	(N = 14)	(N = 16)			
Baseline	57.9 (48.6 to 67.3)	58.8 (50.0 to 67.5)			
Week 24	54.8 (45.5 to 64.2)	57.5 (48.8 to 66.3)			
Δ	−3.08 (−6.92 to 0.76)	−1.24 (−4.83 to 2.35)	−1.92 (−7.04 to 3.19)	0.459	
None or single use	(N = 17)	(N = 17)			
Baseline	59.9 (51.3 to 68.4)	59.6 (51.0 to 68.1)			
Week 24	52.7 (44.2 to 61.3)	56.3 (47.7 to 64.8)			
Δ	−7.16 (−10.61 to −3.71)	−3.28 (−6.73 to 0.17)	−3.85 (−8.61 to 0.92)	0.113	0.935
Dual combination	(N = 24)	(N = 24)			
Baseline	58.6 (51.4 to 65.8)	55.9 (48.7 to 63.1)			
Week 24	54.6 (47.4 to 61.8)	56.2 (49.0 to 63.4)			

Δ	−4.02 (−6.92 to −1.12)	0.34 (−2.56 to 3.24)	−4.10 (−8.11 to −0.09)	0.045	
Triple combination	(N = 8)	(N = 7)			
Baseline	52.5 (40.1 to 65.0)	54.6 (41.3 to 68.0)			
Week 24	49.9 (37.4 to 62.4)	56.1 (42.8 to 69.4)			
Δ	−2.64 (−7.66 to 2.39)	1.46 (−3.92 to 6.83)	−4.29 (−11.47 to 2.90)	0.241	
Serum potassium, mEq/L					
Overall	(N = 49)	(N = 48)			
Baseline	4.2 (4.1 to 4.3)	4.4 (4.3 to 4.5)			
Week 24	4.4 (4.3 to 4.5)	4.3 (4.2 to 4.4)			
Δ	0.22 (0.11 to 0.34)	−0.06 (−0.17 to 0.05)	0.20 (0.06 to 0.33)	0.006	
Use of RASi, No	(N = 9)	(N = 11)			
Baseline	4.1 (3.9 to 4.4)	4.7 (4.5 to 5.0)			
Week 24	4.6 (4.3 to 4.8)	4.4 (4.2 to 4.7)			
Δ	0.44 (0.19 to 0.70)	−0.26 (−0.49 to −0.04)	0.45 (0.14 to 0.76)	0.004	0.071
Use of RASi, Yes	(N = 40)	(N = 37)			
Baseline	4.2 (4.1 to 4.3)	4.3 (4.1 to 4.4)			
Week 24	4.4 (4.2 to 4.5)	4.3 (4.1 to 4.4)			
Δ	0.17 (0.06 to 0.29)	0.00 (−0.12 to 0.12)	0.14 (−0.01 to 0.29)	0.072	
Use of SGLT2i, No	(N = 19)	(N = 17)			0.019

Baseline	4.1 (3.9 to 4.2)	4.3 (4.1 to 4.5)			
Week 24	4.4 (4.2 to 4.6)	4.2 (4.0 to 4.4)			
Δ	0.37 (0.19 to 0.55)	−0.16 (−0.35 to 0.03)	0.41 (0.19 to 0.64)	<0.001	
Use of SGLT2i, Yes	(N = 30)	(N = 31)			
Baseline	4.3 (4.1 to 4.4)	4.4 (4.3 to 4.6)			
Week 24	4.4 (4.2 to 4.5)	4.4 (4.2 to 4.5)			
Δ	0.13 (−0.01 to 0.27)	−0.01 (−0.14 to 0.13)	0.07 (−0.10 to 0.25)	0.405	
Use of GLP-1RA, No	(N = 35)	(N = 32)			
Baseline	4.2 (4.1 to 4.4)	4.3 (4.1 to 4.4)			
Week 24	4.5 (4.3 to 4.6)	4.3 (4.1 to 4.4)			
Δ	0.22 (0.09 to 0.35)	−0.01 (−0.15 to 0.13)	0.20 (0.04 to 0.37)	0.016	0.789
Use of GLP-1RA, Yes	(N = 14)	(N = 16)			
Baseline	4.0 (3.8 to 4.2)	4.5 (4.3 to 4.7)			
Week 24	4.3 (4.1 to 4.5)	4.4 (4.2 to 4.6)			
Δ	0.24 (0.03 to 0.45)	−0.16 (−0.36 to 0.03)	0.16 (−0.09 to 0.42)	0.210	
None or single use	(N = 17)	(N = 17)			
Baseline	4.1 (3.9 to 4.3)	4.4 (4.2 to 4.6)			
Week 24	4.5 (4.3 to 4.7)	4.2 (4.0 to 4.4)			0.022
Δ	0.36 (0.18 to 0.55)	−0.18 (−0.36 to 0.01)	0.43 (0.20 to 0.66)	<0.001	
Dual combination	(N = 24)	(N = 24)			

Baseline	4.2 (4.0 to 4.4)	4.4 (4.2 to 4.5)			
Week 24	4.4 (4.2 to 4.5)	4.4 (4.2 to 4.5)			
Δ	0.15 (−0.01 to 0.31)	0.00 (−0.16 to 0.16)	0.08 (−0.11 to 0.28)	0.400	
Triple combination	(N = 8)	(N = 7)			
Baseline	4.2 (3.9 to 4.5)	4.4 (4.1 to 4.7)			
Week 24	4.3 (4.0 to 4.6)	4.4 (4.1 to 4.7)			
Δ	0.15 (−0.12 to 0.42)	0.01 (−0.28 to 0.31)	0.03 (−0.32 to 0.38)	0.868	
Systolic blood pressure, mm Hg					
Overall	(N = 49)	(N = 48)			
Baseline	132.3 (127.5 to 137.0)	130.0 (125.2 to 134.8)			
Week 24	128.8 (124.1 to 133.6)	133.0 (128.2 to 137.8)			
Δ	−3.45 (−8.27 to 1.37)	3.00 (−1.87 to 7.87)	−5.30 (−11.30 to 0.69)	0.082	
Use of RASi, No	(N = 9)	(N = 11)			
Baseline	139.0 (127.9 to 150.1)	127.4 (117.4 to 137.4)			
Week 24	131.6 (120.5 to 142.6)	135.3 (125.3 to 145.3)			
Δ	−7.44 (−18.76 to 3.87)	7.91 (−2.32 to 18.14)	−9.35 (−22.73 to 4.03)	0.170	0.491
Use of RASi, Yes	(N = 40)	(N = 37)			
Baseline	130.8 (125.5 to 136.0)	130.8 (125.4 to 136.3)			
Week 24	128.2 (123.0 to 133.5)	132.4 (126.9 to 137.8)			

Δ	−2.55 (−7.92 to 2.82)	1.54 (−4.04 to 7.12)	−4.11 (−10.84 to 2.62)	0.230	
Use of SGLT2i, No	(N = 19)	(N = 17)			0.424
Baseline	134.2 (126.7 to 141.7)	132.9 (125.0 to 140.8)			
Week 24	132.9 (125.4 to 140.4)	140.9 (133.0 to 148.8)			
Δ	−1.32 (−9.04 to 6.41)	8.00 (−0.16 to 16.16)	−8.61 (−18.26 to 1.03)	0.080	
Use of SGLT2i, Yes	(N = 30)	(N = 31)			
Baseline	131.1 (125.1 to 137.0)	128.5 (122.6 to 134.3)			
Week 24	126.3 (120.3 to 132.2)	128.7 (122.8 to 134.6)			
Δ	−4.80 (−10.95 to 1.35)	0.26 (−5.79 to 6.30)	−3.67 (−11.08 to 3.73)	0.329	
Use of GLP-1RA, No	(N = 35)	(N = 32)			0.620
Baseline	129.6 (124.0 to 135.2)	129.6 (123.7 to 135.4)			
Week 24	126.6 (121.0 to 132.2)	130.6 (124.7 to 136.4)			
Δ	−3.06 (−8.79 to 2.68)	1.00 (−5.00 to 7.00)	−4.02 (−11.25 to 3.21)	0.274	
Use of GLP-1RA, Yes	(N = 14)	(N = 16)			
Baseline	138.9 (130.1 to 147.8)	130.9 (122.6 to 139.2)			
Week 24	134.5 (125.6 to 143.4)	137.9 (129.6 to 146.2)			
Δ	−4.43 (−13.49 to 4.64)	7.00 (−1.48 to 15.48)	−7.31 (−18.19 to 3.58)	0.187	
None or single use	(N = 17)	(N = 17)			0.438
Baseline	133.8 (125.8 to 141.8)	131.2 (123.2 to 139.2)			
Week 24	131.6 (123.6 to 139.6)	138.3 (130.3 to 146.3)			

Δ	−2.12 (−10.35 to 6.11)	7.06 (−1.17 to 15.29)	−7.84 (−17.85 to 2.16)	0.124	
Dual combination	(N = 24)	(N = 24)			
Baseline	130.0 (123.2 to 136.7)	128.0 (121.2 to 134.7)			
Week 24	126.4 (119.6 to 133.1)	128.1 (121.4 to 134.9)			
Δ	−3.58 (−10.51 to 3.34)	0.17 (−6.76 to 7.09)	−2.69 (−11.11 to 5.72)	0.529	
Triple combination	(N = 8)	(N = 7)			
Baseline	136.1 (124.5 to 147.8)	134.1 (121.7 to 146.6)			
Week 24	130.3 (118.6 to 141.9)	137.0 (124.5 to 149.5)			
Δ	−5.88 (−17.87 to 6.12)	2.86 (−9.97 to 15.68)	−7.69 (−22.77 to 7.40)	0.316	
Diastolic blood pressure, mm Hg					
Overall	(N = 49)	(N = 48)			
Baseline	73.7 (70.3 to 77.2)	74.4 (70.9 to 77.8)			
Week 24	70.9 (67.5 to 74.4)	74.9 (71.4 to 78.4)			
Δ	−2.80 (−6.19 to 0.60)	0.52 (−2.91 to 3.95)	−3.63 (−7.83 to 0.58)	0.090	
Use of RASi, No	(N = 9)	(N = 11)			
Baseline	71.6 (63.5 to 79.6)	72.5 (65.1 to 79.8)			
Week 24	66.6 (58.5 to 74.6)	73.9 (66.6 to 81.2)			0.453
Δ	−5.00 (−12.96 to 2.96)	1.45 (−5.75 to 8.66)	−6.89 (−16.25 to 2.47)	0.148	
Use of RASi, Yes	(N = 40)	(N = 37)			

Baseline	74.2 (70.4 to 78.0)	74.9 (70.9 to 78.9)			
Week 24	71.9 (68.1 to 75.7)	75.2 (71.2 to 79.2)			
Δ	−2.30 (−6.08 to 1.48)	0.24 (−3.68 to 4.17)	−2.89 (−7.64 to 1.86)	0.231	
Use of SGLT2i, No	(N = 19)	(N = 17)			0.508
Baseline	70.7 (65.2 to 76.3)	74.2 (68.4 to 80.1)			
Week 24	69.4 (63.9 to 74.9)	76.8 (70.9 to 82.6)			
Δ	−1.32 (−6.68 to 4.05)	2.53 (−3.15 to 8.21)	−5.50 (−12.35 to 1.35)	0.115	
Use of SGLT2i, Yes	(N = 30)	(N = 31)			
Baseline	75.6 (71.2 to 80.0)	74.4 (70.1 to 78.7)			
Week 24	71.9 (67.5 to 76.3)	73.8 (69.5 to 78.2)			
Δ	−3.73 (−8.01 to 0.54)	−0.58 (−4.78 to 3.62)	−2.59 (−7.84 to 2.65)	0.330	
Use of GLP-1RA, No	(N = 35)	(N = 32)			0.243
Baseline	73.1 (69.1 to 77.2)	75.9 (71.7 to 80.2)			
Week 24	68.3 (64.2 to 72.3)	74.8 (70.5 to 79.0)			
Δ	−4.86 (−8.85 to −0.86)	−1.19 (−5.37 to 2.99)	−5.04 (−10.07 to 0.00)	0.050	
Use of GLP-1RA, Yes	(N = 14)	(N = 16)			
Baseline	75.1 (68.7 to 81.5)	71.2 (65.2 to 77.2)			
Week 24	77.5 (71.1 to 83.9)	75.1 (69.1 to 81.1)			
Δ	2.36 (−3.96 to 8.68)	3.94 (−1.97 to 9.85)	0.35 (−7.17 to 7.88)	0.926	
None or single use	(N = 17)	(N = 17)			0.419

Baseline	73.2 (67.3 to 79.1)	74.1 (68.2 to 80.0)		
Week 24	67.5 (61.6 to 73.4)	74.2 (68.3 to 80.1)		
Δ	−5.76 (−11.55 to 0.02)	0.12 (−5.66 to 5.90)	−6.28 (−13.41 to 0.86)	0.084
Dual combination	(N = 24)	(N = 24)		
Baseline	72.1 (67.2 to 77.1)	74.8 (69.8 to 79.8)		
Week 24	71.7 (66.7 to 76.7)	75.5 (70.6 to 80.5)		
Δ	−0.42 (−5.28 to 4.45)	0.75 (−4.12 to 5.62)	−2.45 (−8.46 to 3.57)	0.423
Triple combination	(N = 8)	(N = 7)		
Baseline	79.5 (70.9 to 88.1)	73.6 (64.4 to 82.8)		
Week 24	75.9 (67.3 to 84.5)	74.3 (65.1 to 83.5)		
Δ	−3.62 (−12.05 to 4.80)	0.71 (−8.30 to 9.72)	−1.49 (−12.29 to 9.31)	0.786

* For group differences of changes from baseline to week 24 in the corresponding variables, the P value for interaction was obtained from the treatment × subgroup interaction term.

eGFR, estimated glomerular filtration rate; GLP-1RA, glucagon-like peptide-1 receptor antagonist; RASi, renin-angiotensin system inhibitor; SGLT2i, sodium-glucose cotransporter 2 inhibitor

Supplementary Reference

S1. Tanaka A, Shibata H, Imai T, Yoshida H, Miyazono M, Takahashi N, Fukuda D, Okada Y, Teragawa H, Suwa S, Kida K, Moroi M, Taguchi I, Toyoda S, Shimabukuro M, Tanabe K, Tanaka K, Nangaku M, Node K; FIVE-STAR trial investigators. Rationale and design of an investigator-initiated, multicenter, prospective, placebo-controlled, double-blind, randomized trial to evaluate the effects of finerenone on vascular stiffness and cardiorenal biomarkers in type 2 diabetes and chronic kidney disease (FIVE-STAR). *Cardiovasc Diabetol*. 2023;22:194.
